# Supplementary material for: Cyclophilin A enables specific HIV-1 Tat palmitoylation and accumulation in uninfected cells
Source: Nat Commun. 2018 Jun 8;9:2251. doi: 10.1038/s41467-018-04674-y (PMC5993824; doi:10.1038/s41467-018-04674-y)
Supplement: Supplementary file 1 — Supplementary Information [file 41467_2018_4674_MOESM1_ESM.pdf]

Supplementary Figure 1

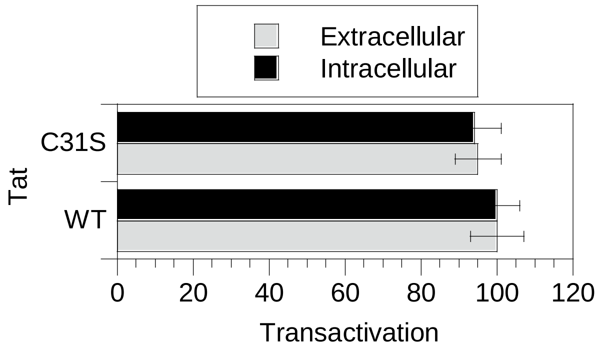

The C31S mutation does not affect Tat capacity to enter cells or its transcriptional activity. PC12 cells were cotransfected with a Tat vector (Intracellular), a vector expressing a Firefly gene under the control of an LTR promoter, a plasmid with a Renilla gene under a CMV promoter and a human cyclin T1 vector. When indicated (Extracellular), cells were not transfected with Tat but were treated with recombinant Tat, WT or C31S for 24 h. Luciferase activities were then assayed and transactivation is expressed as the Firefly/ Renilla ratio (%) compared to the corresponding control (Vendeville et al, 2004). Mean ± SEM, n=4.

Supplementary Figure 2

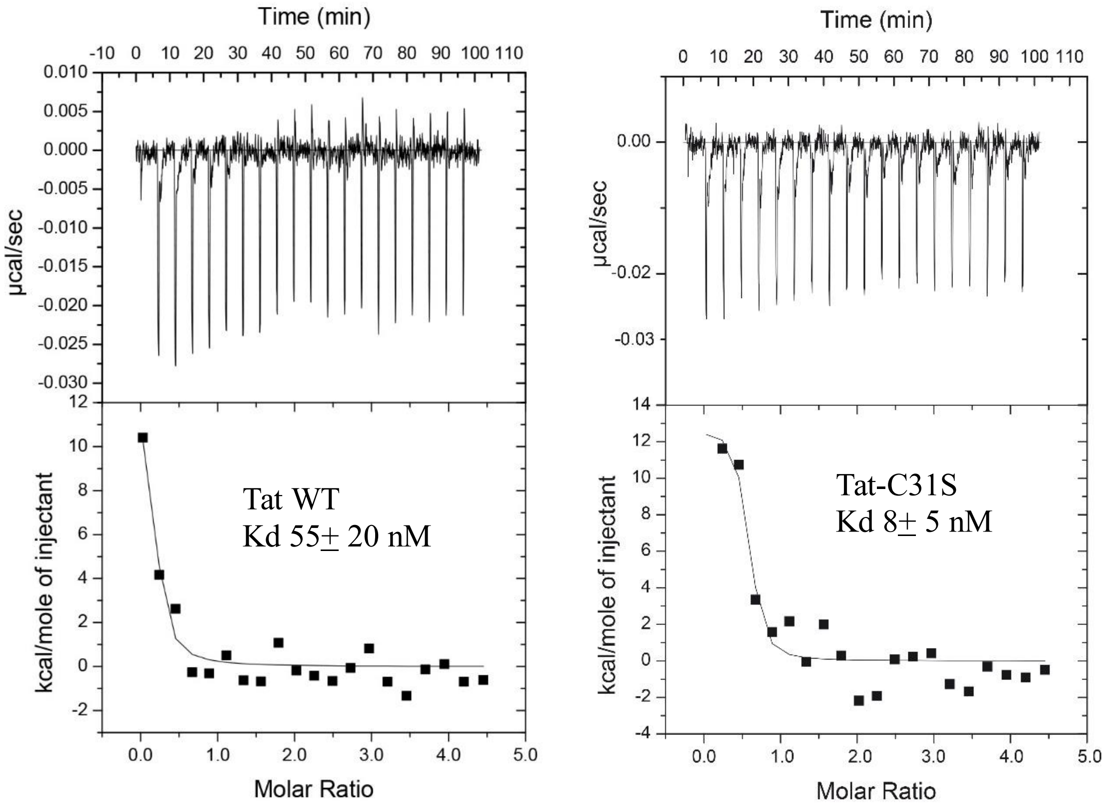

Interaction of Tat-C31S with PI(4,5)P2 liposomes. Raw ITC data. Liposomes (PC/PG/PI(4,5)P2 75/20/5; 200 µM) were injected into 1 µM Tat WT or C31S (1µM) in 150 mM NaCl, 50 mM Citrate, pH 7.3, and heat production was monitored. The heat resulting from buffer injection was subtracted from the binding curves. The Tat-PI(4,5)P_2 liposome interaction in NaCl buffer is exothermic (DH<0), while in KCl buffer it is endothermic (DH>0)[1]. Mean ± SEM (n=3).

Supplementary Figure 3

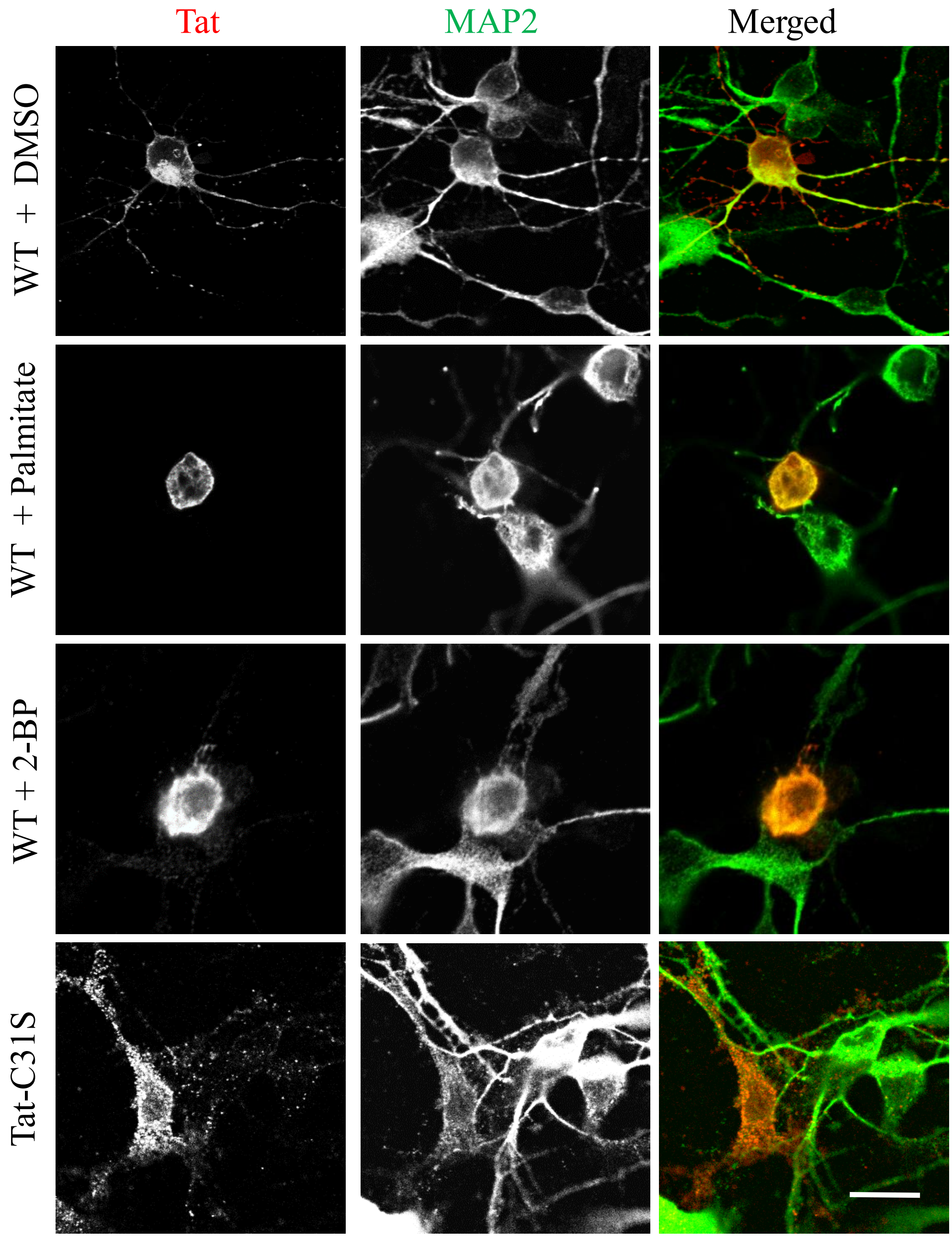

Tat palmitoylation is required for membrane association in primary neurons. Hippocampal neurons were transfected with a Tat vector. After 20h, when indicated, 50 µM 2-BP, palmitate or solvent was added for 4 h. Neurons were then fixed for Tat and MAP2 (to confirm neuronal identity) immunostaining then imaged using a confocal microscope. Representative median optical sections are shown. Bar, 10 µm. n=10.

Supplementary Figure 4

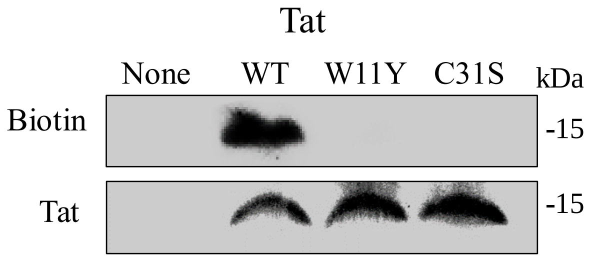

Exogenous Tat-C31S and Tat-W11Y are not palmitoylated. Jurkat cells were treated overnight with 100 µM 17-ODYA and 50 nM Tat-His$_6$, WT, C31S or W11Y as indicated. After cell lysis, Tat-His$_6$ was isolated on Ni-NTA-agarose beads before click chemistry to attach biotin to 17-ODYA, Western blot and detection of biotin then Tat.

Supplementary Figure 5

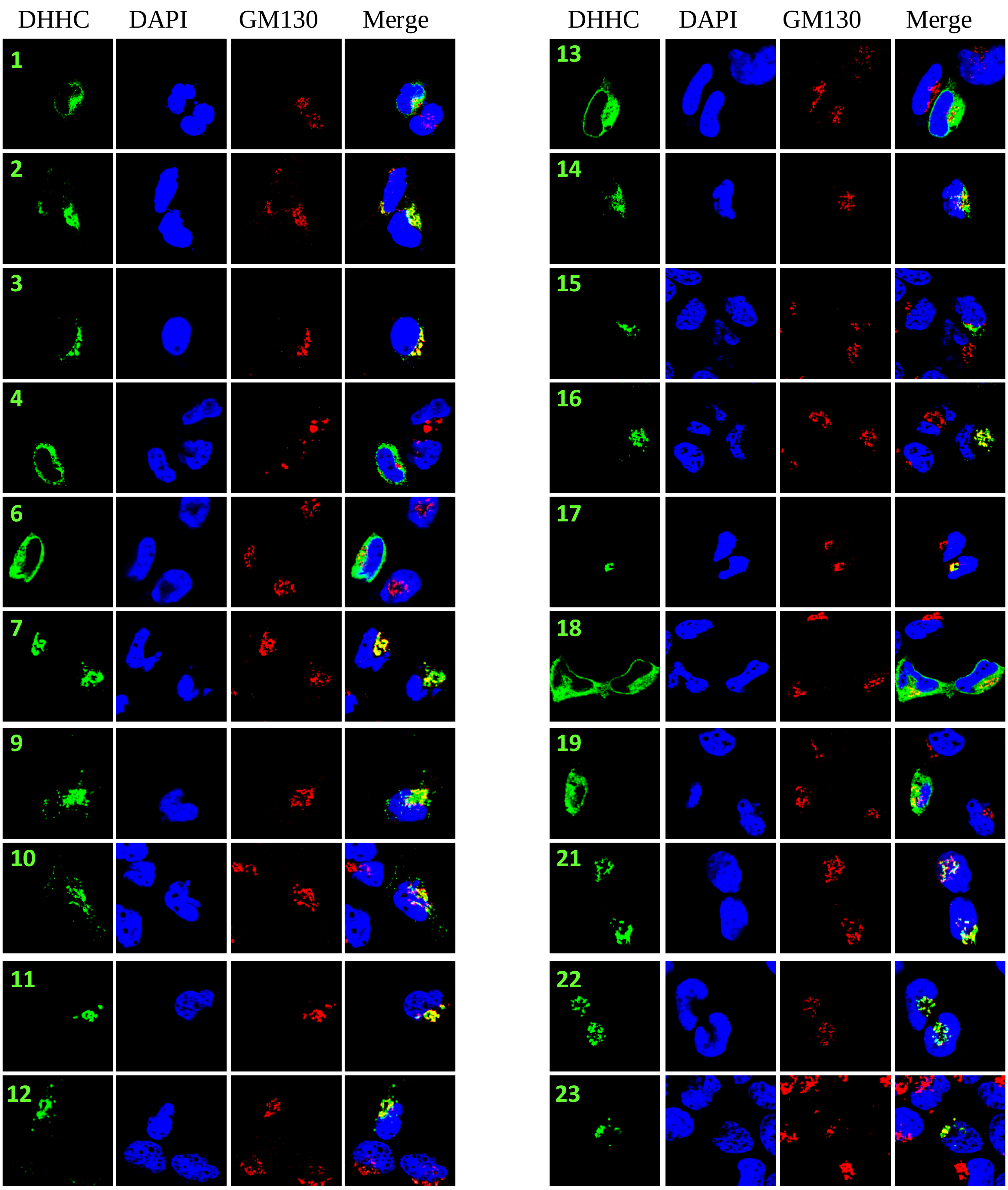

Localization of DHHC enzymes in PC12 cells. Cells were transfected with the indicated GFP-tagged mouse DHHC vectors. They were then fixed and permeabilized for GM130 immunolabelling and nuclei staining using DAPI, or plasma membrane and Golgi labelling using fluorescent wheat germ agglutinnin (WGA). Cells were then examined by confocal microscopy. Representative median optical sections are shown. Bar, 10 µm.

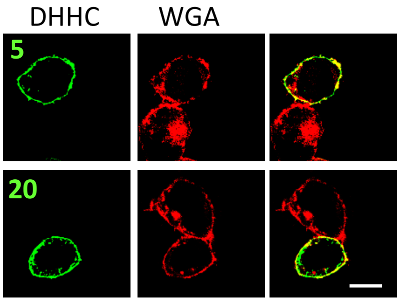

Supplementary Figure 6

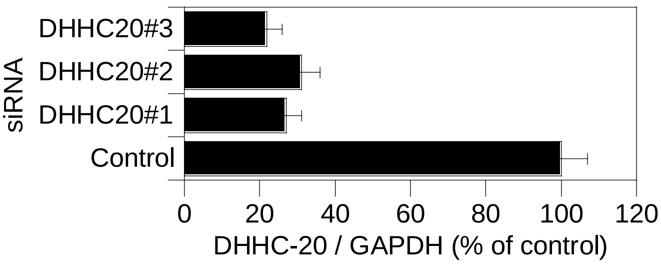

Extinction of DHHC-20 expression using siRNAs. PC12 cells were transfected with the indicated siRNA before RNA extraction and qRT-PCR to quantify DHHC-20 and GAPDH mRNAs. Results are expressed as percentages of the DHHC-20 / GAPDH control ratio. DHHC-20 siRNA #3 was used for palmitoylation experiments (Fig.3c). Means ± SEM, n=3.

Supplementary Figure 7

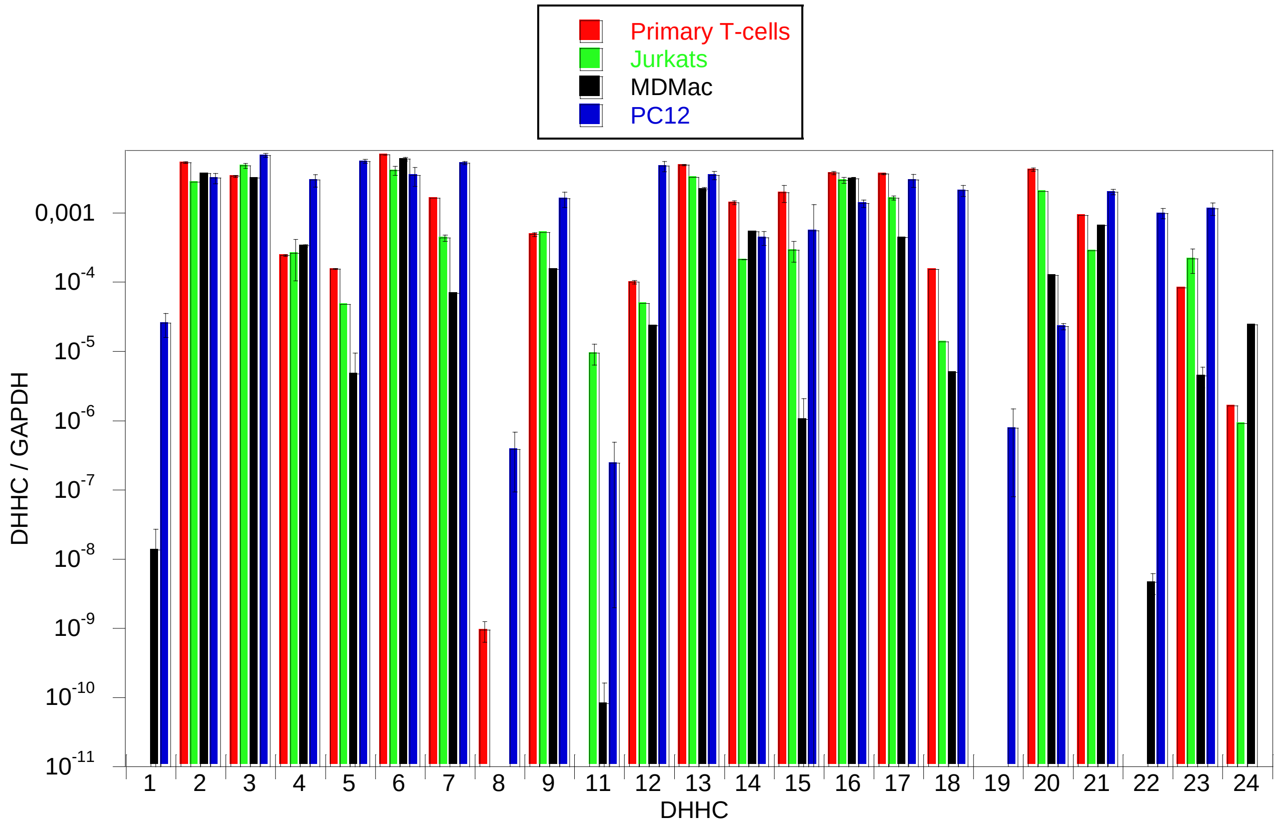

DHHC expression levels in macrophages, neuron precursors and T-cells. The RNA from monocyte-derived primary macrophages (MDMAc), PC12, Jurkat or human primary $CD4^+$ T-cells was extracted before quantification of DHHCs and GAPDH mRNAs using qRT-PCR. Results are expressed as DHHC / GAPDH ratio. There are 23 DHHCs in mammals; DHHC-10 does not exist[2]. An absent bar indicates that the corresponding DHHC was not detectable in this cell type. Means ± SEM, n=3.

Supplementary Figure 8

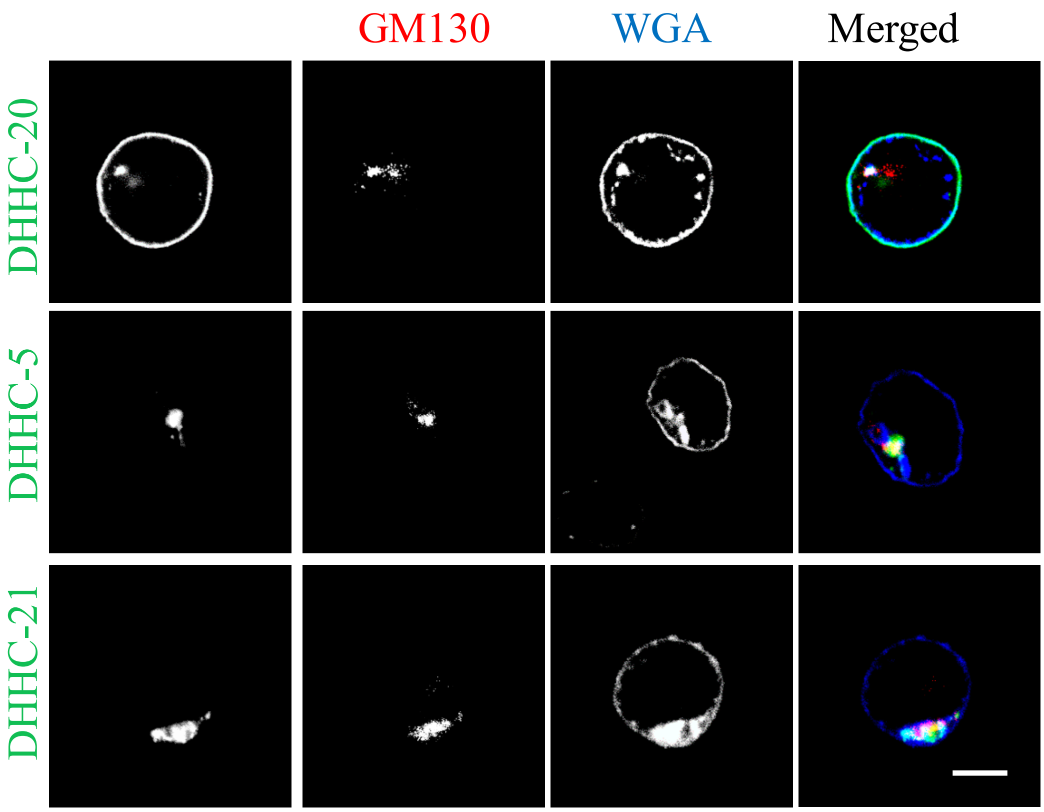

Localization of DHHC-GFP in human primary T-cells. Cells were transfected with the indicated GFP-tagged mouse DHHC enzyme then fixed and permeabilized for GM130 immunolabelling to localize the Golgi apparatus, and plasma membrane and Golgi labelling using fluorescent wheat germ agglutinin. They were then examined by confocal microscopy. Representative median optical sections are shown. Bar, 5 µm.

Supplementary Figure 9

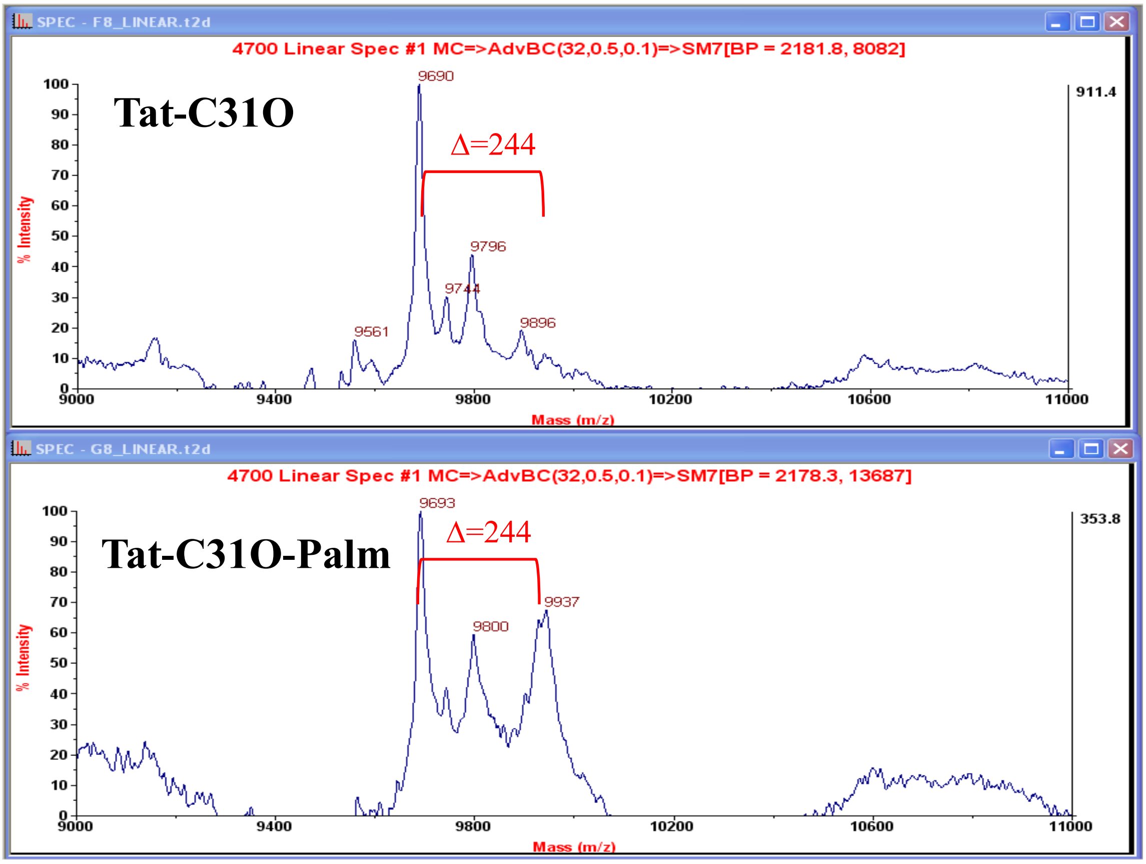

Purity and palmitoylation efficiency of Tat-C31O. The theoretical MW of protonated Tat-C31O (MEPVDPRLEPWKHPGSQPKTASTNSYSKKSCFHSQVSFITKALGISYGRKKRRQRRRPPQGSQTHQVSLSKQPTSQSRGDPTGPKE) is 9689. Upon palmitate addition, an H2O is lost and a theoretical mass increase of 238 should be observed. Analysis performed using a 4800 Plus MALDI-TOF/TOF Proteomics Analyser showed that a Δmass of 244 (± 3 Da, the precision of the instrument) is observed, confirming that Tat-C31O was palmitoylated. The peak-area ratio indicates that palmitoylation took place with an efficacy of 47 ± 8%.

Supplementary Figure 10

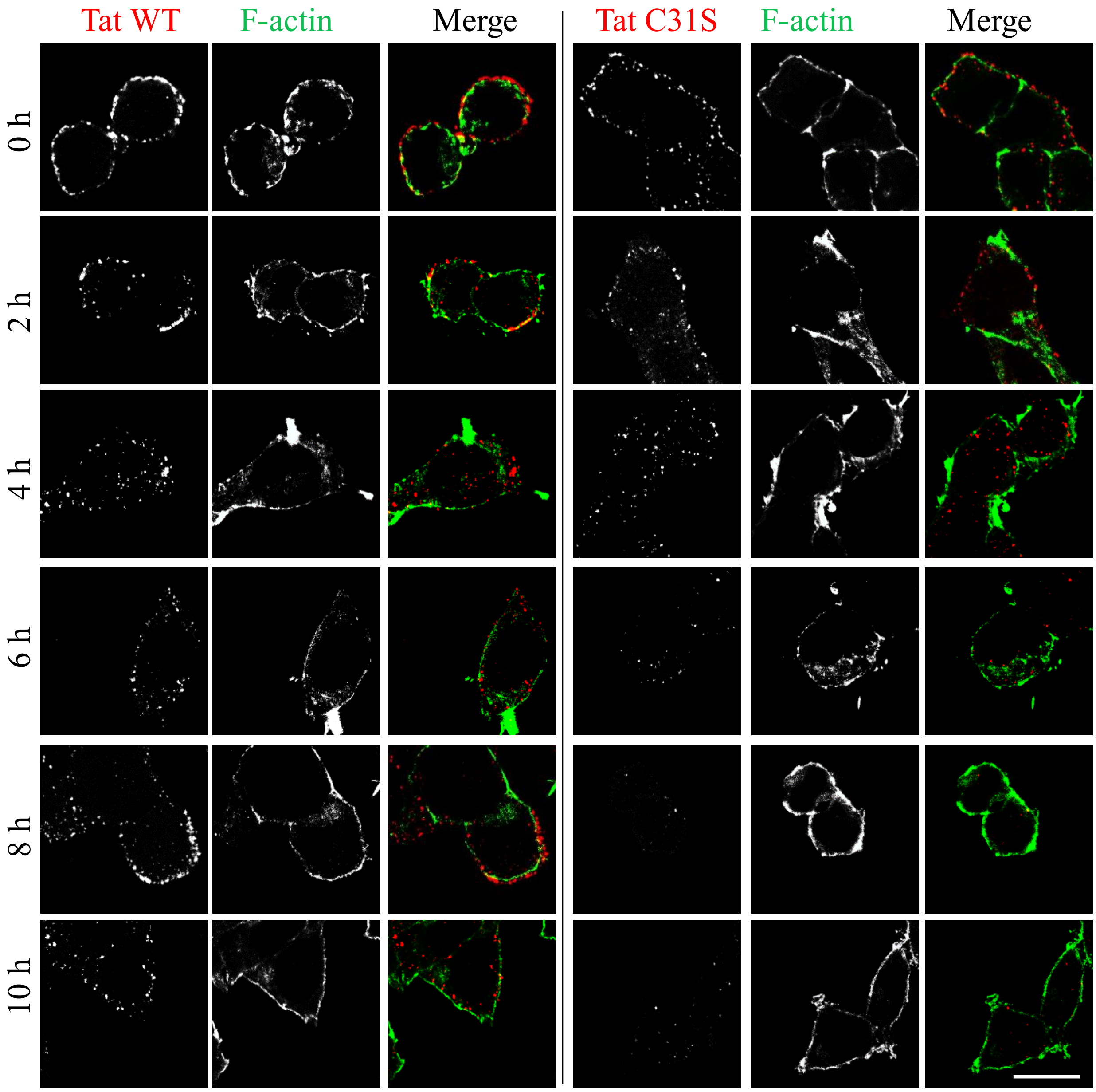

Palmitoylation is required for the recruitement at the plasma membrane of incoming Tat. PC12 cells were labeled with 100 nM Tat (WT or C31S) for 30 min at 4°C. After washing, cells were incubated at 37°C and fixed after the indicated times for Tat staining by immunofluorescence and F-actin labeling using fluorescent phalloidin. Representative median optical sections obtained with a confocal microscope are shown. Bar, 10 µm. Images from $50 < n < 100$ cells were used to quantify Tat / F-actin colocalization using Manders' coefficient (Figure 4B).

Supplementary Figure 11

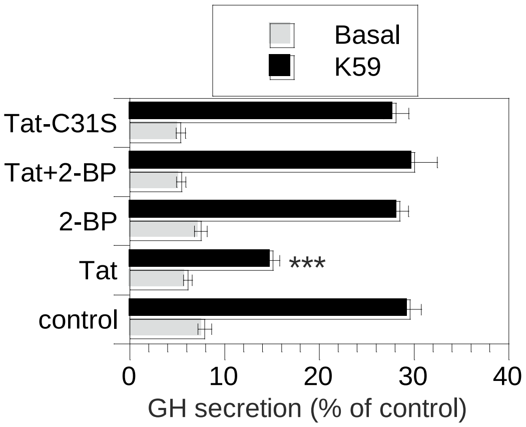

Palmitoylation is required for Tat to inhibit neurosecretion by chromaffin cells. Cells were transfected with human growth hormone (GH), then treated with 20 nM Tat (WT or C31S) and/or 100 µM 2-BP for 5 h as indicated. They were then incubated for 10 min in calcium-free Locke's solution (basal release) or stimulated for 10 min with a depolarizing concentration of K^+ (K59; Locke's solution containing 59 mm KCl). GH secretion was then assayed by ELISA. Mean ± SEM (n=3). Data were analyzed using a two-way ANOVA (***, $p<0.001$).

Supplementary Figure 12

a

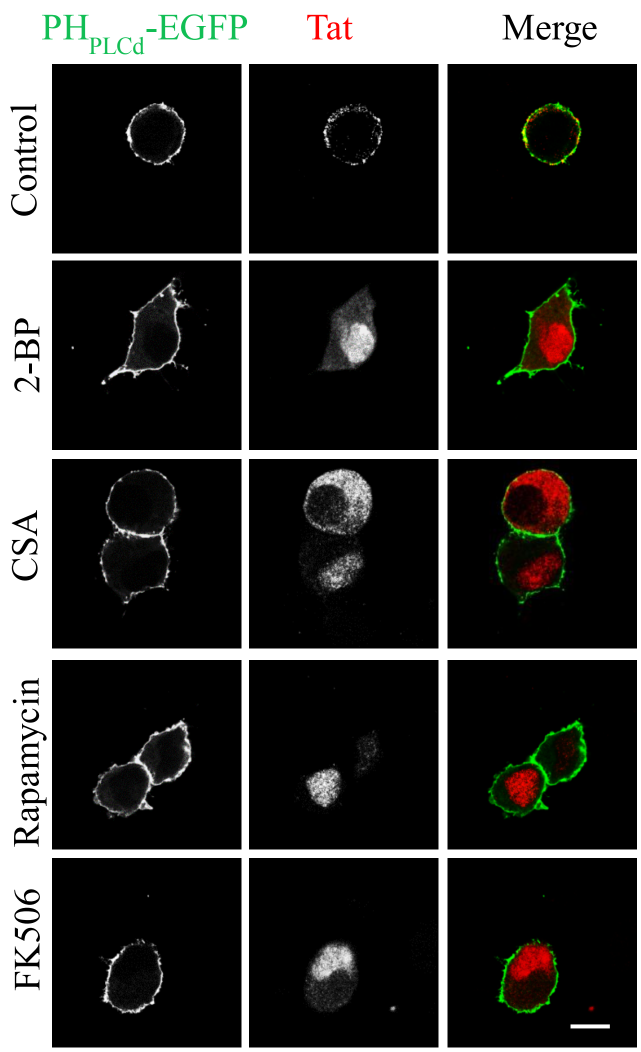

Immunophilin inhibitors displace Tat to the cytosol. PC12 cells were cotransfected with Tat and an EGFP chimera of the PLCδ-PH. After 24 h they were treated for 5 h with the indicated drugs (100 µM 2-BP; 2 µM CSA; 1 µM rapamycin; 1 µM FK506), then washed and fixed for Tat staining by immunofluorescence. Representative median optical sections obtained with a confocal microscope. Bar, 10 µm.

b

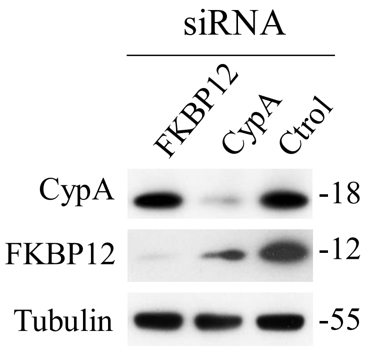

Silencing FKBP12 and CypA in Jurkat cells .Cells were transfected with the indicated siRNA. After 24 h they were lysed for Western blotting.
Silencing CypA also affected FKBP12 expression.

Supplementary Figure 13

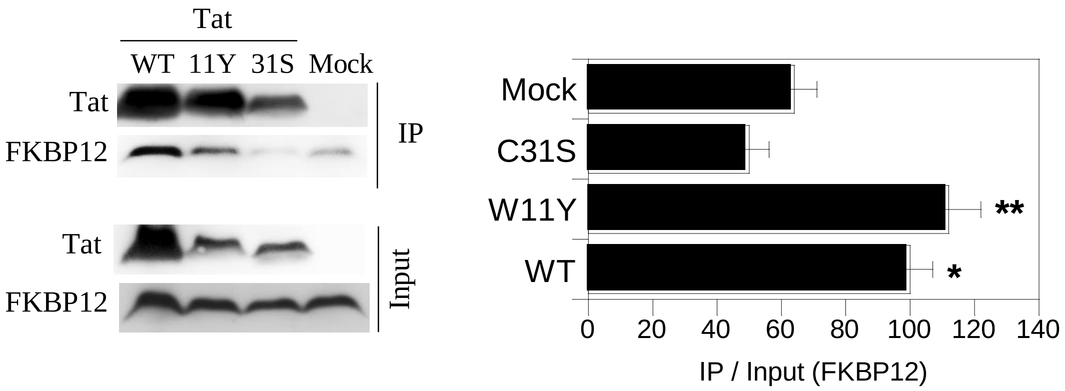

HIV-1 Tat interacts with FKBP12. HEK 293T cells were transfected with an empty vector (mock) or pBi-Tat-FLAG (WT, 11Y or C31S). Cells were lysed 24 h after transfection and Tat-FLAG was immunoprecipitated before anti-FKBP12 Western blotting. The graph shows the quantification from triplicate blots. Data (mean ±SEM, n=3) were analysed using one-way ANOVA (**, p<0.01; *, p<0,05).

Supplementary Figure 14

a

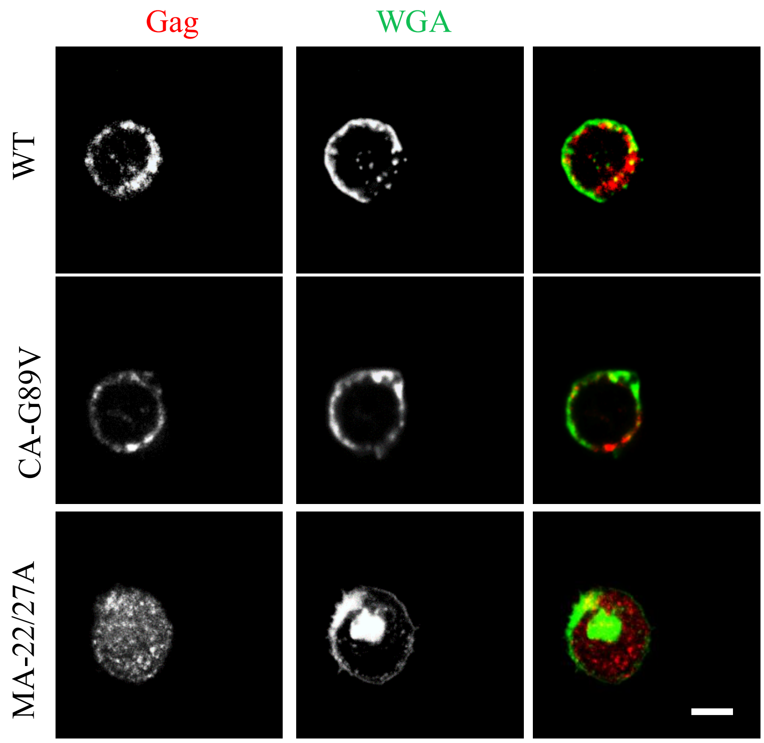

Localisation of Gag mutants in human primary T-cells. CD4+ primary T-cells were transfected with the indicated Gag mutant then fixed and permeabilized for p24 immunolabelling, and plasma membrane and Golgi labelling using fluorescent wheat germ agglutinin (WGA). They were then examined by confocal microscopy. Representative median optical sections are shown. Bar, 5 µm.

b

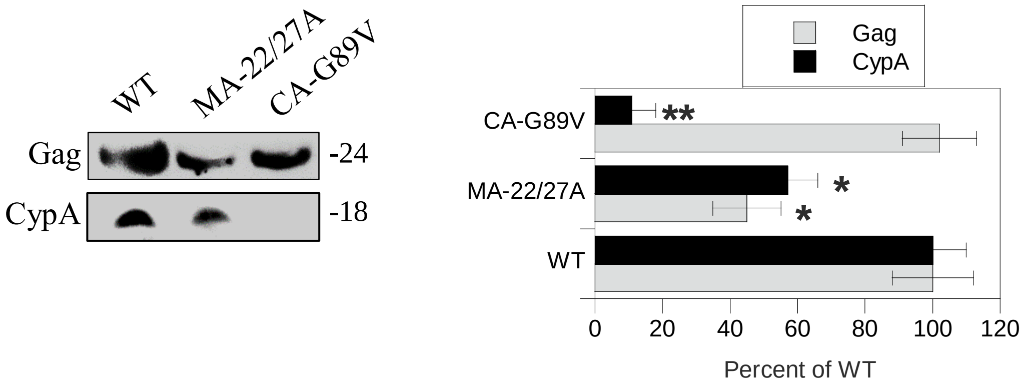

VLP production from mutant Gag proteins. Jurkat T-cells were transfected with the indicated Gag mutant; 48 h later, the medium was filtered and VLP were isolated by ultracentrifugation and analysed by western blotting. Quantification corresponds to 3 different experiments. Mean ± s.e.m. Data were analyzed using a one way ANOVA (**, $p<0.01$; *, $p < 0.05$).

Supplementary Figure 15

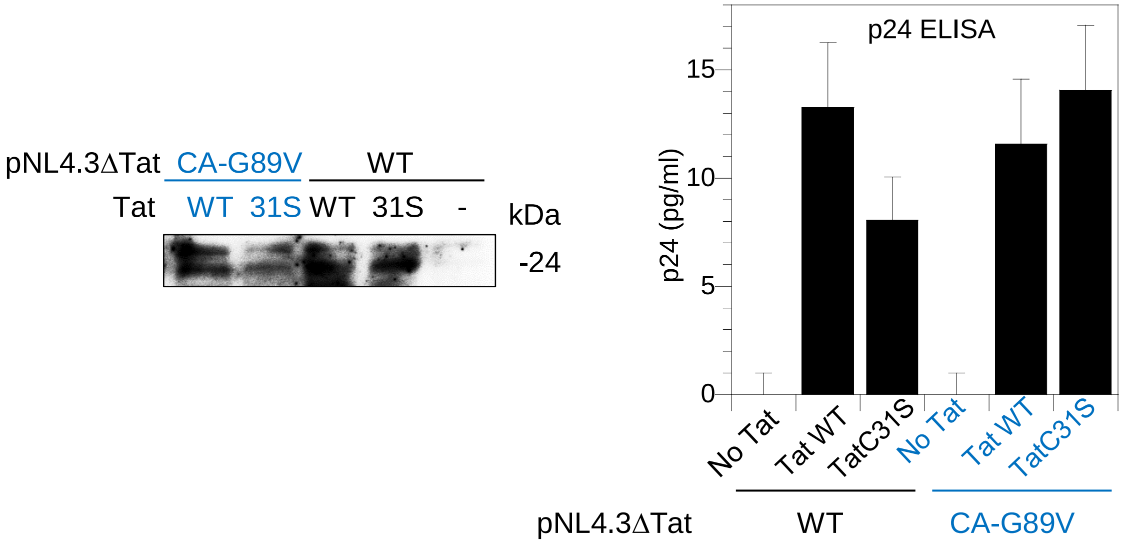

Complementation of pNL4.3ΔTat by pBi-Tat-FLAG enables virus production. HEK 293 T cells were cotransfected with pNL4.3ΔTat, WT or CA-G89V and pBi-Tat-FLAG (WT or C31S) using a ratio 4 pNL4.3 / 1 Tat. Viruses were purified from the cell medium 48 h after transfection and were analysed by SDS/PAGE and anti HIV-1 p24 Western blotting or p24 ELISA (mean ± SEM, n=3).

Supplementary Figure 16

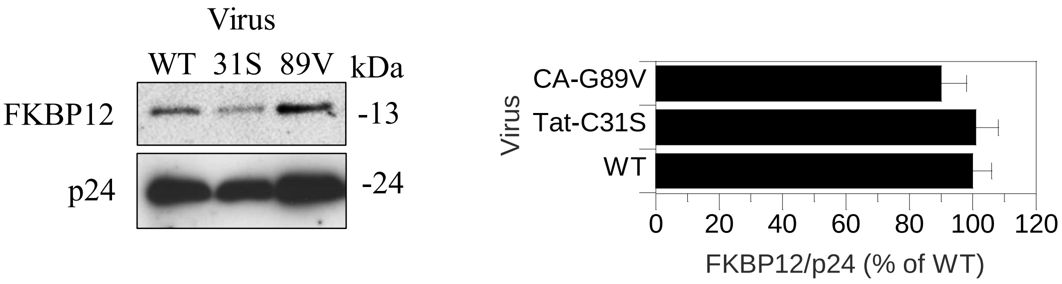

Levels of FKBP12 in WT, Tat-C31S and CA-G89V viruses. Jurkat CD4 T-cells were infected with HIV-1 (NL4.3) either WT, Tat-C31S or CA-G89V. Viruses were purified from the cell medium 48 h after infection before anti-FKBP12 and anti HIV-1 p24 Western blots. Representative experiment from n=3 experiments. The graph presents the FKBP12 levels normalized using p24 amounts (mean ± SEM).

Supplementary Figure 17

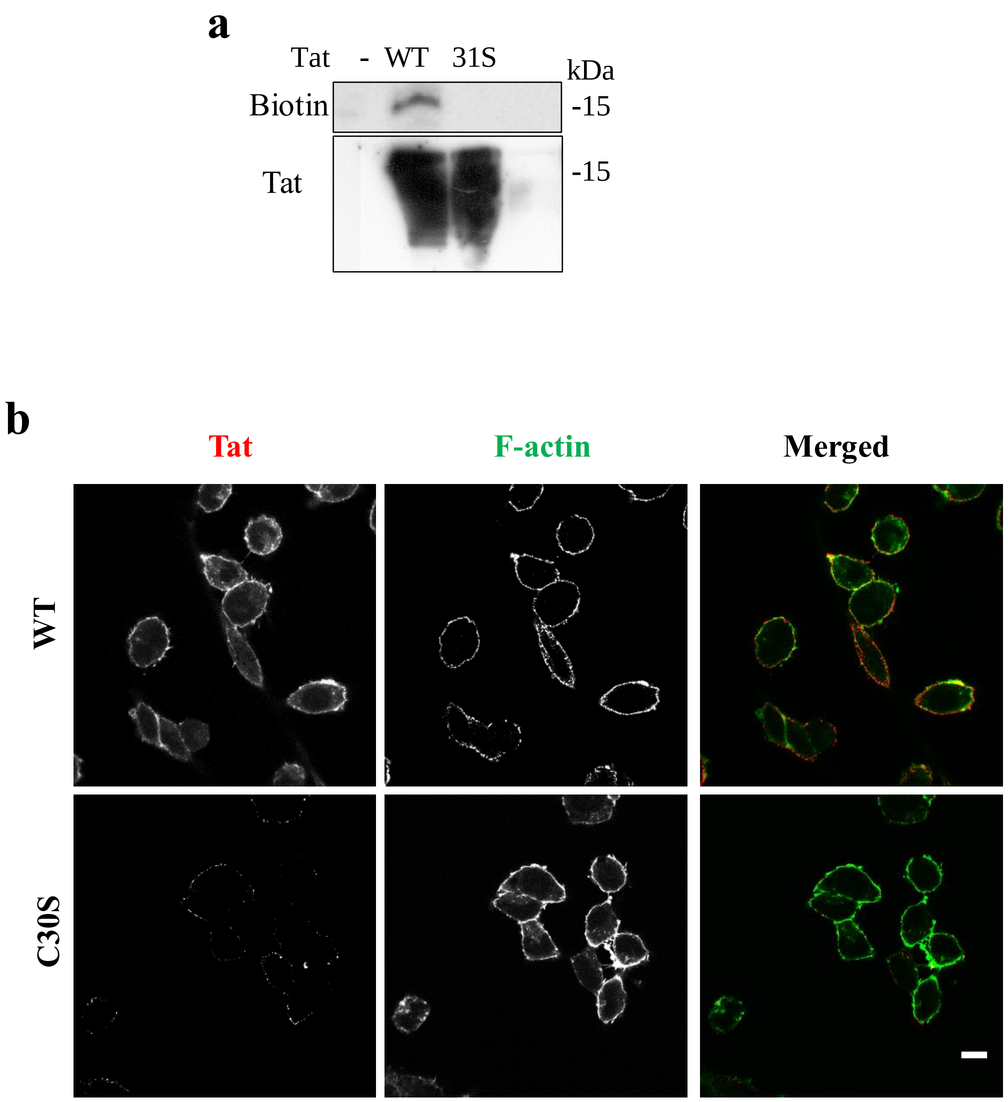

Exogenous Tat-C30S is not palmitoylated because it is not transported to the cytosol. a PC12 cells were treated overnight with 100 µM 17-ODYA and 50 nM Tat-His$_6$ WT, C30S, or C31S as indicated. After cell lysis, Tat-His$_6$ was isolated on Ni-NTA-agarose beads before click chemistry to attach biotin to 17-ODYA, Western blot and detection of biotin then Tat. WT Tat only was palmitoylated and Tat-C30S was not detectable even on overexposed blots. **b** Tat (100 nM; WT or C30S) was pre-bound to cells on ice before washes, fixation, Tat staining by immunoflorescence and imaging. Bar, 10 µm. Quantification of 50<n<60 cells showed that the amount of cell-associated Tat-C30S was decreased by 57± 3% compared to WT Tat.

Supplementary Figure 18, Uncropped blots

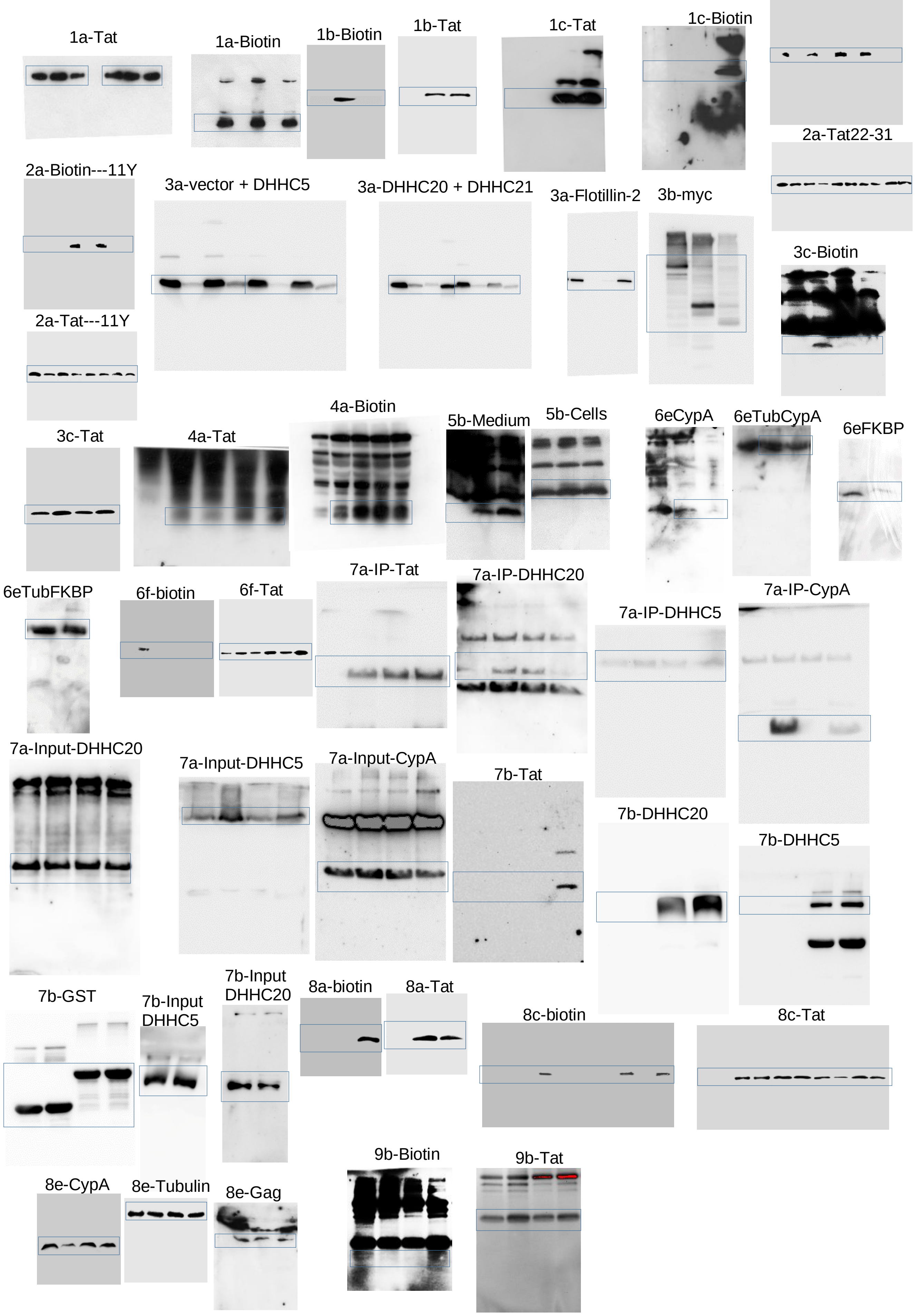

Supplementary Table 1.

B	B Ions	B+2H	B-NH3	B-H2O	AA	Y Ions	Y+2H	Y-NH3	Y-H2O	Y
1	171.1	86.1	154.1		K+42	1,954.0	977.5	1,937.0	1,936.0	13
2	331.1	166.1	314.1		C+57	1,783.9	892.4	1,766.9	1,765.9	12
3	672.4	336.7	655.4		C+238	1,623.8	812.4	1,606.8	1,605.8	11
4	819.5	410.2	802.4		F	1,282.6	641.8	1,265.6	1,264.6	10
5	956.5	478.8	939.5		H	1,135.5	568.3	1,118.5	1,117.5	9
6	1,059.5	530.3	1,042.5		C	998.5	499.7	981.5	980.5	8
7	1,187.6	594.3	1,170.6		Q	895.5	448.2	878.4	877.5	7
8	1,286.6	643.8	1,269.6		V	767.4	384.2	750.4	749.4	6
9	1,446.7	723.8	1,429.6		C+57	668.3	334.7	651.3	650.3	5
10	1,593.7	797.4	1,576.7		F	508.3	254.7	491.3	490.3	4
11	1,706.8	853.9	1,689.8		I	361.2	181.1	344.2	343.2	3
12	1,807.9	904.4	1,790.9	1,789.9	T	248.2	124.6	231.1	230.1	2
13	1,954.0	977.5	1,937.0	1,936.0	K	147.1	74.1	130.1		1

Fragmentation table of HIV-1 Tat peptide 29-41 (KCCFHCQVCFITK). PC12 cells were transfected with Tat-FLAG before immunoprecipitation using anti-FLAG antibodies, SDS/ PAGE , excision of the gel band and analysis by nano LC-MS/MS. The C+238 B and Y ions indicate that Tat is palmitoylated on Cys 31, while the others Cys are modified by carbamidomethylation (C+ 57 Da).
Sequence coverage (50%) includes all Cys residues and no modification of the other Cys was observed.

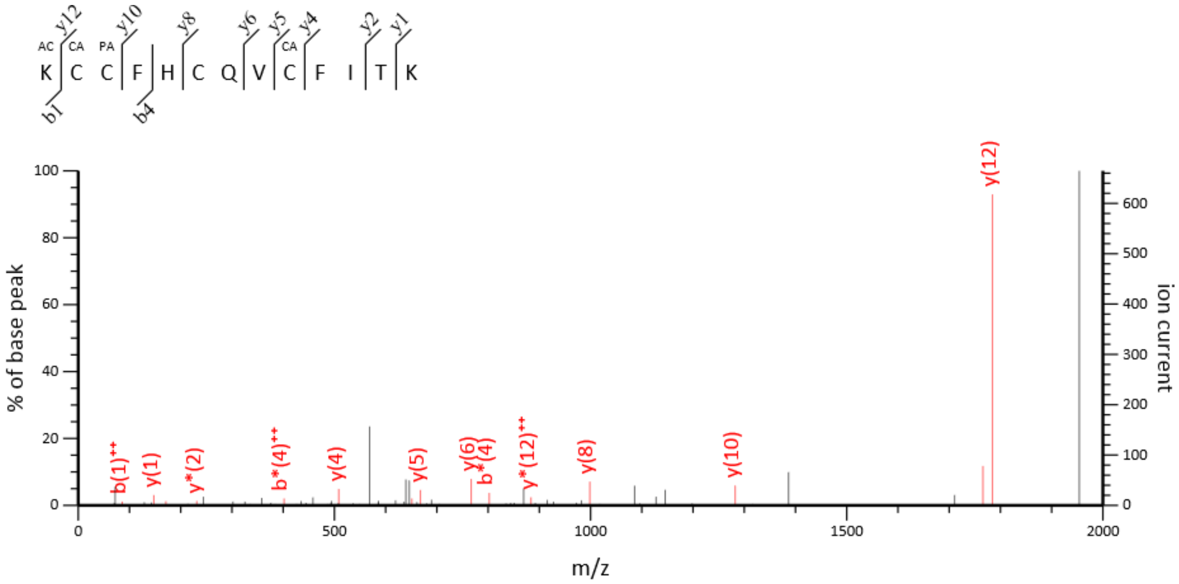

Fragmentation spectrum of HIV-1 Tat peptide 29-41 (KCCFHCQVCFITK)

Supplementary Table 2

DHHC primers used for real-time PCR

	**For human DHHCs**	**For rat and mouse DHHCs**
**DHHC1 sense**	GCGCAGCTGTTTTCAAGTCA	TTGCATGGGTGCCATCTTTG
**DHHC1 antisense**	CCACACACTCTTCTCAGGGG	TAGCTCTTGTCCCGCACATT
**DHHC2 sense**	TGGAGACAGAGCCAAGAGGAG	
**DHHC2 antisense**	TTATAAGTTGGCATCTGTCACAGTATC	
**DHHC3 sense**	ACCCACCACAGTGATTCTCC	TGTCACCTGGTTTCTGGTCC
**DHHC3 antisense**	GTGGCCAAAAACGGCTTTCA	GTCTCGGGATGGAATCAGCA
**DHHC4 sense**	TGTGGAACCAATCCTGGCAT	TAACCCCACAGTGCTTCCAG
**DHHC4 antisense**	ACAGTGATGGTCGAAACGGT	TTCCGCGTAAACTAGCCCTT
**DHHC5 sense**	GAAGACTGAAGAAAGATAAGAGACATTC	
**DHHC5 antisense**	GACACTTCAAAAGTTTACTGTGGATG	
**DHHC6 sense**	AGCACCACTGGGTTGTATCC	GCAACCCGAGGGTTACGATA
**DHHC6 antisense**	ACTGTGTTCCACCCAAAGGA	TCCATCACAGGGGCACTTTT
**DHHC7 sense**	CAGTTCATCTCCTGTGTCCGA	CGTGGGTGAACAACTGTGTG
**DHHC7 antisense**	GGCTTCTCACTTTTCAATCGCT	AAGGCACAGGAAGACCAACA
**DHHC8 sense**	ACTCCCCACTCACCATCCTA	ATGCTCTTGTGGGAGGTGATG
**DHHC8 antisense**	TGCAGGTGCCAATCCGC	ACTCGGTATGCTTTGGGGTG
**DHHC9 sense**	GTGCTGGATCGAAGGGGTAT	GAAACTCCCGGGCAGGAATA
**DHHC9 antisense**	TGTCCTCCGGCATCTCATTT	ACAGCCAGATAGCGACACTC
**DHHC11 sense**	GTCGTTACCCCTGCACTACT	CCTCCACTCCTTTCAGGCAA
**DHHC11 antisense**	GGTGGAACGAGAAGATCCCC	AACATGAACACCCCACCCAT
**DHHC12 sense**	GTGCTCTTCCTGCACGATAC	GCATGGTCTGGTCTCCAGTT
**DHHC12 antisense**	ACATTCACGTAGCCAGGGTC	GGCGATTCGGTGTGAGGATA
**DHHC13 sense**	TGAAACAGACGTTGTCCCTCA	TCACTGGGCTGCCATTAACA
**DHHC13 antisense**	TGTCCAATCTACCACACAGGG	CAAATGCCCTTGTCGGATGG
**DHHC14 sense**	CATCGTTGGCCTCTCAGGAT	GAGGCTATATCCAGCCCGAC
**DHHC14 antisense**	GTCGATCAGGCTTGGTGAGA	CGCTCTGAAGTAGGGGAGTG
**DHHC15 sense**	TATGAATGAGTCACAGAACCCACTGCTAG	
**DHHC15 antisense**	TAATCTTGGTTGTCATCCTCGTTGTCTTCC	
**DHHC16 sense**	TGGGTGCCCTAACTGTATGG	CTAAGCCAGCACGAACACAC
**DHHC16 antisense**	AATACTCTGCCCTTGGCCTG	ATTGTTTAGCCAGGGGCAGT
**DHHC17 sense**	GGTGAACCTCTTGGACGGAA	TTACGACAACCCGTCCTTCC
**DHHC17 antisense**	TTTTCTTTGTCCGGTTGCCG	AGCCCAAACACCACCATACA
**DHHC18 sense**	CAGTGTCTGCGACAACTGTG	GGGTTCCACACTTACCTCGT
**DHHC18 antisense**	GAAGGCCGTCAGGAATGAGA	ATGGCTGTAGGGGTTGACAG
**DHHC19 sense**	AGTGGCCTCTTCTTCGCATT	CAAAGTGCCTCTTCCATCGC
**DHHC19 antisense**	CTCAGCGGAGCCTTGATGTA	GACCGATGCAGTTGTTGACC
**DHHC20 sense**	ATCAACCTTTTCCTATCAAACCACTTAG	

**DHHC20 antisense**	GCCTTCTTCAGCTCCATTCTCC	
**DHHC21 sense**	TCTGGGAATTATGTAACAAGTGTAATTG	GGACCTCTTTGTTGTTCGGC
**DHHC21 antisense**	AGAGCCAATGATTATCTTCACCAAC	GGAACTCTCAGTGGTTGCCT
**DHHC22 sense**	ATGCTCTACCTCTGGTTCGC	GGCACGACCATDACTGTTTC
**DHHC22 antisense**	AGCCACCTCTTTCCGAAGAC	GAGATATAGGCCACTCCGGC
**DHHC23 sense**	TGGCTTAGGGGAGCCAAAAA	ATAAACAGCTGCGTCGGAGA
**DHHC23 antisense**	AGTAGTACCACAGTGCCAGC	ACGCAGGTGAAGGATAGAGC
**DHHC24 sense**	CTGGCTCATGTTGCTCACAG	The DHHC24 gene is absent in mouse cells.
**DHHC24 antisense**	GGACCCAGGTCATAGGAGTG	
**GAPDH sense**	ACCACGAGAAATATGACAACTCC	
**GAPDH antisense**	CCAAAGTTGTCATGGATGACC	

Supplementary References

1. Rayne F*, et al.* Phosphatidylinositol-(4,5)-bisphosphate enables efficient secretion of HIV-1 Tat by infected T-cells. *EMBO J* **29**, 1348-1362 (2010).

2. Greaves J, Chamberlain LH. DHHC palmitoyl transferases: substrate interactions and (patho)physiology. *Trends Biochem Sci* **36**, 245-253 (2011).
